# Supplementary material for: Anti-Inflammatory Effect of Synaptamide in Ischemic Acute Kidney Injury and the Role of G-Protein-Coupled Receptor 110
Source: Int J Mol Sci. 2024 Jan 25;25(3):1500. doi: 10.3390/ijms25031500 (PMC10855239; doi:10.3390/ijms25031500)
Supplement: Supplementary file 1 [file ijms-25-01500-s001.zip › ijms-2807319-supplementary.pdf]

Anti-Inflammatory Effect of Synaptamide in Ischemic Acute Kidney Injury and the Role of G-Protein-Coupled Receptor 110

Anna A. Brezgunova [1,2], Nadezda V. Andrianova [1], Aleena A. Saidova [3], Daria M. Potashnikova [3], Polina A. Abramicheva [1], Vasily N. Manskikh [1], Sofia S. Mariasina [4,5,6], Irina B. Pevzner [1,7], Ljubava D. Zorova [1,7], Igor V. Manzhulo [8], Dmitry B. Zorov [1,7] and Egor Y. Plotnikov [1,7,*]

[1] A.N. Belozersky Institute of Physico-Chemical Biology, Lomonosov Moscow State University, 119992 Moscow, Russia; brezgunova.anna.2014@post.bio.msu.ru (A.A.B.); andrianova@belozersky.msu.ru (N.V.A.); abramicheva.polina@belozersky.msu.ru (P.A.A.); manskikh@mail.ru (V.N.M.); pevzner_ib@belozersky.msu.ru (I.B.P.); ljuzor@belozersky.msu.ru (L.D.Z.); zorov@belozersky.msu.ru (D.B.Z.)

[2] Faculty of Bioengineering and Bioinformatics, Lomonosov Moscow State University, 119992 Moscow, Russia

[3] Faculty of Biology, Lomonosov Moscow State University, 119991 Moscow, Russia; aleena.saidova@gmail.com (A.A.S.); dpotashnikova@gmail.com (D.M.P.)

[4] Department of Chemistry, Lomonosov Moscow State University, 119991 Moscow, Russia; sofia.mariasina@yandex.ru

[5] Faculty of Fundamental Medicine, Lomonosov Moscow State University, 119991 Moscow, Russia

[6] Research and Educational Resource Center "Pharmacy", RUDN University, 117198 Moscow, Russia

[7] V.I. Kulakov National Medical Research Center for Obstetrics, Gynecology and Perinatology, Ministry of Healthcare of Russian Federation, 117198 Moscow, Russia

[8] A.V. Zhirmunsky National Scientific Center of Marine Biology, Far Eastern Branch, Russian Academy of Sciences, 690041 Vladivostok, Russia; i-manzhulo@bk.ru

* Correspondence: plotnikov@belozersky.msu.ru; Tel.: +7-495-939-5944

Supplementary Figures

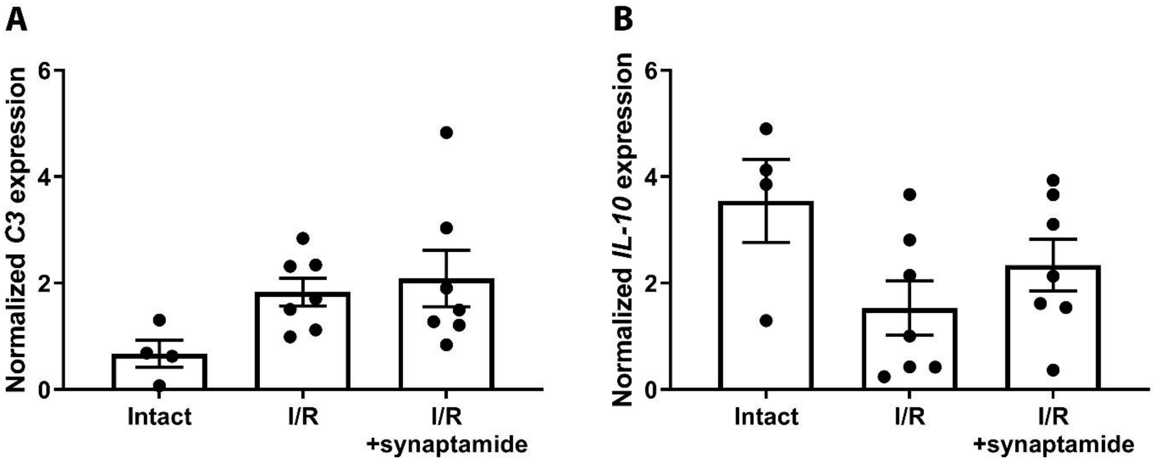

Supplementary Figure S1. Expression of inflammatory mediators after renal I/R and synaptamide treatment. (**A**) *C3* mRNA expression. (**B**) *IL-10* mRNA expression.

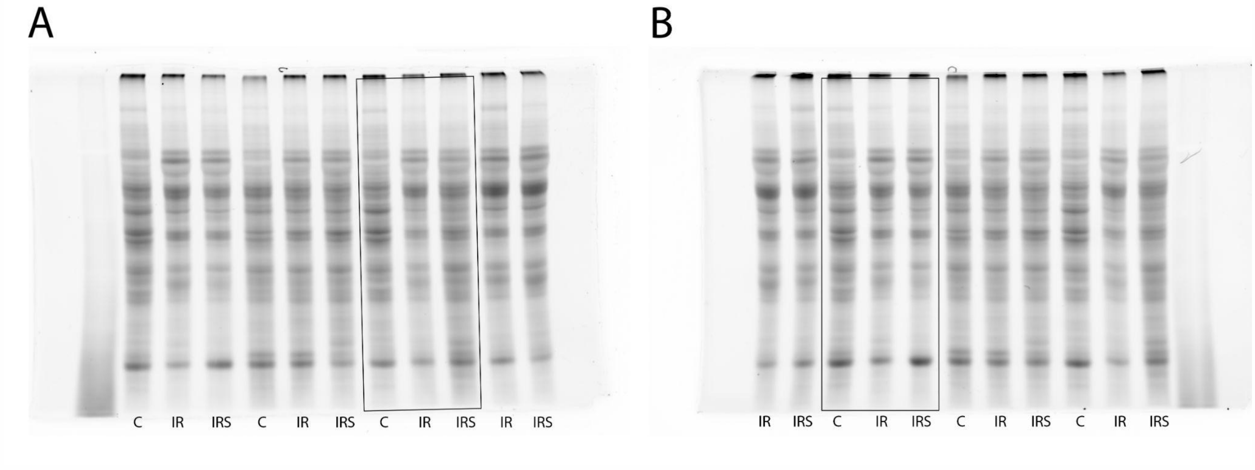

Supplementary Figure S2. Validation of total protein normalization. Stain-free imaging of kidney homogenate samples for Figure 1E (**A**) and for Figure 1F (**B**).
